# Cardiac Hemodynamics are Linked With Structural and Functional Features of Brain Aging: The Age, Gene/Environment Susceptibility (AGES)‐Reykjavik Study

**DOI:** 10.1161/JAHA.114.001294

**Published:** 2015-01-27

**Authors:** Behnam Sabayan, Mark A. van Buchem, Sigurdur Sigurdsson, Qian Zhang, Tamara B. Harris, Vilmundur Gudnason, Andrew E. Arai, Lenore J. Launer

**Affiliations:** Department of Radiology, Leiden University Medical Center, Leiden, The Netherlands (B.S., M.A.B.); Icelandic Heart Association, Reykjavik, Iceland (S.S., V.G.); Intramural Research Program, National Institute on Aging, National Institutes of Health, Bethesda, MD (Q.Z., T.B.H., L.J.L.); Cardiovascular and Pulmonary Branch, National Heart, Lung, and Blood Institute, National Institutes of Health, Bethesda, MD (A.E.A.)

**Keywords:** brain aging, cardiac output, cognitive impairment, stroke volume

## Abstract

**Background:**

Advanced heart failure is linked with structural and functional alterations in the brain. It is unclear whether a graded decrease in cardiac function puts older subjects at risk for brain aging. We investigated the association between cardiac hemodynamics and features of brain aging in community‐dwelling older subjects.

**Methods and Results:**

With data from a sub‐study (n=931 subjects, mean age 75.9 years, 47.7% male) of the Age, Gene/Environment Susceptibility (AGES)‐Reykjavik Study, we investigated the association of MRI measures of cardiac hemodynamics, including left ventricular stroke volume (LVSV) and cardiac output (CO) to brain characteristics. In multivariable analyses, each 10 mL lower LVSV was associated with 4.4 mL (95% CI 1.9 to 6.9) lower total parenchymal brain volume (TBV) and 3.7 mL (95% CI 1.8 to 5.7) lower gray matter volume (GMV). Likewise, each unit (L/min) lower CO was associated with 3.9 mL (95% CI 0.4 to 7.4) lower TBV and 3.9 mL (95% CI 0.4 to 7.4) lower GMV. Lower LVSV was associated with worse performance in processing speed (*P*=0.043) and executive function (*P*<0.001). Lower CO was associated with worse performance in processing speed (*P*=0.015) and executive function (*P*=0.003). Each 10 mL lower LVSV and each unit lower CO associated with a higher risk of mild cognitive impairment or dementia (odds ratio: 1.24, 95% CI 0.99 to 1.57 and odds ratio: 1.40, 95% CI 0.99 to 2.00, respectively).

**Conclusions:**

A graded decrease in cardiac functioning is associated with features of brain aging**.** Older persons with cardiac or cognitive signs and symptoms may have both cardiac and cerebral diseases and should be evaluated accordingly.

## Introduction

Structural and functional integrity of the brain depends on adequate and constant supply of oxygen and nutrients through cerebral blood flow.^1^ Chronic cerebral hypoperfusion has been shown to play a key role in development of age‐related brain pathologies in animal models.^2^ Animal models with decreased cerebral blood flow carry a higher risk for neuronal injury and death, blood‐brain barrier disruption, cerebrovascular pathologies, and cognitive deficit.^3–6^

Congestive heart failure has a well‐established association with decline in cerebral blood flow.^7^ Hence, patients with heart failure have been described as human models for studying the impact of cerebral hypoperfusion on abnormal brain aging.^8^ Different lines of evidence indicate that patients with heart failure are at a higher risk for structural and functional brain abnormalities and interventions to improve cardiac functioning might lead to neurocognitive benefits in these patients.^9–11^ Recently, it has been hypothesized that early disturbances in cardiac functioning, as reflected in cardiac hemodynamics, should also be considered as a risk factor for abnormal brain aging.^8^ A few studies investigated this hypothesis, however the interpretation of their findings is hampered because of methodological limitations such as assessment of brain outcomes years before cardiac measurements or evaluating cardiac function with parameters that only measure systolic function of the heart.^12–13^ In addition, these studies were mainly conducted in middle age or young old people. Given the scarcity of comprehensive data on the link between suboptimal changes in cardiac functioning and features of brain aging in community‐dwelling older subjects, we aimed to investigate the association of cardiac hemodynamics with structural and functional features of brain aging in a community‐based cohort of older subjects.

## Methods

### Study Population

Participants were from the ICELAND MI (Imaging Cardiac Evaluation to Locate Areas of Necrosis and Detect MI) study, which is a sub‐study of the Age, Gene/Environment Susceptibility (AGES)–Reykjavik Study (n=5764).^14^ The AGES–Reykjavik study is a population‐based cohort of men and women born between 1907 and 1935 in Iceland. This study was approved by the National Bioethics Committee in Iceland that acts as the institutional review board for the Icelandic Heart Association and by the National Institute on Aging intramural institutional review board. Participants in the AGES–Reykjavik study were enrolled from 2002 to 2006. Details of inclusion procedure for the AGES–Reykjavik study have been reported previously.^15^ To be eligible for the sub‐study participants should be free of contraindications for MRI scanning and gadolinium contrast injection. All participants gave informed consent for this sub‐study.

The overall study goals and study design of the ICELAND MI study have been described previously.^14^ Participants in the ICELAND MI cohort were recruited in 2 phases. The first phase involved random recruitment and a second phase recruited all eligible and willing participants with diabetes. For the phase 1, 839 individuals were invited and 702 enrolled. In the second phase, all 421 eligible participants with diabetes were invited and 290 people enrolled. Of the enrolled subjects, 34 participants had non‐diagnostic cardiac MRI scans due to arrhythmia or inability to hold their breath (n=14), claustrophobia (n=7), inability to gate cardiac images (n=3), technical issues with reconstruction and data transfer (n=9), or artifacts from spinal implants (n=1). For 5 participants data on the brain outcomes were not available leaving a final cohort of 931 participants (including 671 from phase 1 and 260 from phase 2) for this analysis.

### Cardiac MRI and Hemodynamic Parameters

Cardiac magnetic resonance scans were performed on a study‐dedicated 1.5‐T Signa Twinspeed system (GE Healthcare) using a 4‐element cardiac‐phased array coil. Typical cine steady‐state free precession scan parameters resulted in pixel dimensions of 1.8×2.1 mm, a slice thickness of 8 mm with a 3‐mm gap, and 30 images per cycle. Standard long‐axis and short‐axis views were obtained to evaluate global and regional function. End‐systolic phase was determined as the minimal cross‐sectional area of a midventricular slice. Left ventricular end diastolic volume (LVEDV) and left ventricular end systolic volume (LVESV) were computed by the summation of disks method. Left ventricular stroke volume (LVSV) in mL was calculated by subtracting LVESV from LVEDV. Left ventricular ejection fraction (LVEF) in % was calculated as LVSV/LVED×100 and cardiac output (CO) in L/min was calculated as LVSV/1000× heartbeat per minute.

### Brain Imaging

All the participants underwent a high‐resolution brain MRI on the same 1.5‐T system. The imaging protocol has been described previously^15–16^ and included 3D spoiled‐gradient recalled T1‐weighted, fast spin echo proton density/T2‐weighted, fluid‐attenuated inversion recovery (FLAIR) and echo‐planar imaging gradient echo T2*‐weighted sequences. All images were acquired to give full brain coverage with slices angled parallel to the anterior commissure‐posterior commissure line in order to give reproducible image views in the oblique‐axial plane. Total brain, white and grey matter, and white matter hyperintensity volumes were computed automatically with the AGES‐Reykjavik/Montreal Neurological Institute pipeline, which accommodates full brain coverage including cerebellum and brainstem, includes multispectral images (T1‐weighted 3D spoiled‐gradient recalled sequence, FLAIR and proton density/T2‐weighted fast spin echo sequences), and is high throughput and with minimal editing. Data on manifestations of cerebral small vessel disease including white matter hyperintensities, infarcts, and cerebral microbleeds (CMB) were obtained. The segmentation pipeline, its components and accuracy has been described in detail elsewhere.^17^ White matter hyperintensities were considered present in regions where signal intensity was higher than that of normal white and grey matter on both T2‐weighted and FLAIR images. Infarcts and CMB were evaluated qualitatively according to a standardized protocol that required a neuroradiologist to identify the parenchymal defects and radiographers who entered additional data on slice, location, and image characteristics. Infarcts were defined as a defect of the brain parenchyma with signal intensity equal to cerebrospinal fluid on all pulse sequences (FLAIR, T2‐weighted, proton density weighted). CMB were defined as focal areas of signal void within the brain parenchyma that (1) are visible on T2*‐weighted GRE‐EPI images, (2) are smaller or invisible on T2‐weighted FSE images (“blooming effect”), (3) are not abutting a parenchymal defect, and (4) do not show any other structure in the area of signal void.

### Cognitive Function and Depressive Symptoms

A battery of 6 different neurocognitive tests was administered to all participants. From these tests, 3 cognitive domain composite scores were calculated: (1) the memory composite score included the immediate and delayed recall of a modified version of the California Verbal Learning Test^18^; (2) the speed of processing composite included the Figure Comparison Test,^19^ the Digit Symbol Substitution Test,^20^ and the Stroop Test^21^ part 1 and 2; and (3) the executive function composite included a short version of the Cambridge Neuropsychological Test Automated Battery Spatial Working Memory test, the Digits Backward test,^20^ and the Stroop test part 3. Composite measures were computed by converting raw scores on each test to standardized Z‐scores and averaging the Z‐scores across the tests in each composite. Inter‐rater reliability for all tests was excellent (Spearman correlations range, 0.96 to 0.99). Cognitive impairment was considered to be present if subjects had either mild cognitive impairment or dementia. Mild cognitive impairment and dementia case ascertainment was a 3‐step process described previously.^22^ Briefly, all subjects were screened on cognitive function with the Mini‐Mental State Examination^23^ and Digit Symbol Substitution Test. Those with positive screen results were administered a diagnostic battery of neuropsychological tests and, among them, those with positive screen results were examined by a neurologist and a proxy interview was administered. A consensus diagnosis, according to international guidelines, was made by a panel that included a geriatrician, a neurologist, a neuropsychologist, and a neuroradiologist. Depressive symptoms were assessed using the 15‐item Geriatrics Depression Scale (GDS‐15).^24^ The GDS‐15 is a well‐established screening tool to detect depression symptomology in older persons and scores of 6 points and higher is considered to have high depression symptomology.^25^

### Other Covariates

Level of education and smoking status were assessed by questionnaire. Diabetes was defined as a history of diabetes, use of glucose‐modifying medication, or fasting blood glucose of 7 mmol/L. Hypertension was defined as measured systolic blood pressure 140 mm Hg or diastolic blood pressure 90 mm Hg, or self‐reported doctor's diagnosis of hypertension, or using antihypertensive medications. History of stroke was recorded using questionnaires and medical reports. Prevalent coronary heart disease was defined as self‐reported history of coronary artery disease or coronary artery bypass surgery or angioplasty or angina pectoris on the Rose Angina Questionnaire, hospital records or evidence on electrocardiogram (ECG) of possible or probable myocardial infarction. The diagnosis of atrial fibrillation was made by a twelve lead ECG performed during the AGES‐Reykjavik study comprehensive examination based on Minnesota code 8‐3‐1. Additionally, hospital discharge diagnosis codes from all hospitals in Reykjavik from January 1987 until the day of the study examination were reviewed for the diagnosis of atrial fibrillation (ICD‐9 code 427.9 or ICD‐10 code I48).

### Statistical Analyses

Characteristics of the study participants are reported as mean (standard deviation) for continuous variables and number (percentage) for categorical variables. GDS score is reported as median (interquartile range) since it was not distributed normally. The association between hemodynamic parameters and continuous outcomes was estimated with linear regression models, and with logistic regression models when the outcomes were dichotomous. We performed our analyses in 2 steps. In the first step all the analyses were adjusted for age and sex. In the second step, all analyses were adjusted for age, sex, as well as the other potential confounders^26^ including education, smoking status, hypertension, diabetes mellitus, prevalent coronary heart disease, stroke, total cholesterol, body mass index, atrial fibrillation, and systolic blood pressure. For the association between hemodynamic parameters and brain volumes, analyses were additionally adjusted for intracranial volume. To account for the over‐sampling of diabetics that resulted from our 2‐stage sampling (first phase random selection and in the second phase persons with diabetes), we applied inverse probability weighting method. This method adds a weighting variable to the statistical models to address the issue that subjects with diabetes were over‐represented in the sample. All the probability values were calculated using cardiac hemodynamics as continuous variables. Analysis of covariance was used to calculate the adjusted means and standard errors for the brain volumes and cognitive scores in tertiles of hemodynamic parameters. All analyses were conducted using SAS statistical software, version 9.3 (SAS Institute, Cary, NC).

## Results

Mean age of participants was 75.9 years and 47.7% of them were male. Average values for LVEF, LVSV and CO were 60%, 62 mL, and 4 L/min, respectively (Table 1). Descriptive data for those randomly selected and those with diabetes are provided in Table 2.

**Table 1.** Characteristics of the Study Participants: AGES‐RS Imaging Cardiac Evaluation to Locate Areas of Necrosis and Detect MI (ICELAND‐MI)

Characteristics	Values
Number of participants	931
Socio‐demographics	
Age, mean (SD)	75.9 (5.2)
Male, n (%)	444 (47.7)
Low education*, n (%)	206 (22.2)
Current smoker, n (%)	105 (11.3)
Cardiovascular risk factors	
Hypertension, n (%)	770 (82.7)
Diabetes mellitus, n (%)	330 (35.4)
Coronary heart disease, n (%)	208 (22.3)
Atrial fibrillation, n (%)	138 (14.8)
Systolic blood pressure, mm Hg, mean (SD)	143 (19.7)
Diastolic blood pressure, mm Hg, mean (SD)	74 (9.5)
Total cholesterol, mmol/L, mean (SD)	5.5 (1.2)
Body mass index, kg/m^2^, mean (SD)	27.6 (4.3)
Cardiac hemodynamics	
Left ventricular end diastolic volume, mL, mean (SD)	105 (29.8)
Left ventricular end systolic volume, mL, mean (SD)	43 (23.3)
Left ventricular ejection fraction, %, mean (SD)	60 (9.8)
Left ventricular stroke volume, mL, mean (SD)	62 (15.2)
Cardiac output*, L/min, mean (SD)	4 (1.0)
Brain measures	
Total brain tissue volume, mL, mean (SD)	1082.0 (104.8)
Grey matter volume, mL, mean (SD)	678.0 (64.8)
White matter volume, mL, mean (SD)	384.3 (48.2)
Memory composite score, point, mean (SD)	0.07 (0.9)
Processing speed composite score, point, mean (SD)	0.10 (0.8)
Executive function composite score, point, mean (SD)	0.04 (0.7)
Geriatric depression scale score, point, median (IQR)	2 (1 to 3)
Cognitive impairment*, n (%)	81 (8.8)
White matter lesion volume, mL, mean (SD)	19.7 (18.2)
Infarcts, n (%)	311 (33.4)
Microbleeds, n (%)	110 (11.9)

AGES‐RS indicates age, gene/environment susceptibility‐Reykjavik Study; IQR, inter quartile range; MI, myocardial infarction; n, number; SD, standard deviation.

* Primary school education or less.

* Data for cardiac output was available for 922 participants.

* Presence of mild cognitive impairment or dementia.

**Table 2.** Characteristics of the Study Participants in 2 Groups of Diabetics and Randomly Selected Subjects: AGES‐RS Imaging Cardiac Evaluation to Locate Areas of Necrosis and Detect MI (ICELAND‐MI)

Characteristics	Diabetics	Randomly Selected
Number of participants	260	671
Socio‐demographics		
Age, mean (SD)	75.1 (4.8)	76.2 (5.4)
Male, n (%)	124 (47.7)	308 (45.9)
Low education*, n (%)	53 (20.5)	153 (22.8)
Current smoker, n (%)	28 (10.8)	77 (11.5)
Cardiovascular risk factors		
Hypertension, n (%)	238 (91.5)	532 (79.3)
Diabetes, n (%)	260 (100.0)	70 (10.4)
Coronary heart disease, n (%)	65 (25.0)	143 (21.3)
Atrial fibrillation, n (%)	35 (13.5)	103 (15.4)
Systolic blood pressure, mm Hg, mean (SD)	147.1 (20.5)	141.9 (19.1)
Diastolic blood pressure, mm Hg, mean (SD)	74.6 (9.6)	73.7 (9.5)
Total cholesterol, mmol/L mean (SD)	5.2 (1.2)	5.6 (1.2)
Body mass index, kg/m^2^, mean (SD)	28.9 (4.3)	27.0 (4.2)
Cardiac hemodynamics		
Left ventricular end diastolic volume, mL, mean (SD)	107.3 (33.1)	104.2 (28.4)
Left ventricular end systolic volume, mL, mean (SD)	44.7 (28.0)	42.4 (21.1)
Left ventricular ejection fraction, %, mean (SD)	60.2 (11.4)	60.6 (9.1)
Left ventricular stroke volume, mL, mean (SD)	62.6 (17.0)	61.9 (14.5)
Cardiac output*, L/min, mean (SD)	4.0 (1.0)	4.0 (1.0)
Brain outcomes		
Total brain tissue volume, mL, mean (SD)	1081.5 (107.8)	1082.2 (103.7)
Grey matter volume, mL, mean (SD)	680.8 (67.5)	676.9 (63.9)
White matter volume, mL, mean (SD)	379.8 (47.6)	386.1 (48.3)
Memory composite score, point, mean (SD)	0.13 (0.8)	0.05 (0.9)
Processing speed composite score, point, mean (SD)	0.08 (0.8)	0.11 (0.7)
Executive function composite score, point, mean (SD)	0.04 (0.7)	0.04 (0.7)
Geriatric depression scale score, point, median (IQR)	2 (1 to 3)	2 (1 to 3)
Cognitive impairment*, n (%)	19 (7.5)	62 (9.3)
White matter lesion volume, mL, mean (SD)	21.0 (19.3)	19.2 (17.7)
Infarcts, n (%)	104 (40.0)	207 (30.8)
Microbleeds, n (%)	35 (13.6)	75 (11.2)

AGES‐RS indicates age, gene/environment susceptibility‐Reykjavik Study; IQR, inter quartile range; MI, myocardial infarction; n, number; SD, standard deviation.

* Primary school education or less.

* Data for cardiac output was available for 922 participants.

* Presence of mild cognitive impairment or dementia.

As presented in Table 3, multivariable analyses showed that each 10 mL lower LVSV was associated with 4.4 mL (95% CI 1.9 to 6.9) lower total brain parenchymal volume and 3.7 mL (95% CI 1.8 to 5.7) lower gray matter volume. Likewise, each unit (L/min) lower CO was associated with 3.9 mL (95% CI 0.4 to 7.4) lower total brain parenchymal volume and 3.9 mL (95% CI 1.2 to 6.7) lower gray matter volume. Lower LVSV and CO were not associated with white matter volume and we found no association between LVEF and any of the brain volumes we examined (all *P*>0.05). Adjusted mean values of the brain volumes in tertiles of LVEF, LVSV, and CO are presented in Table 4.

**Table 3.** Association of Cardiac Hemodynamic Measures With Brain Volumes: AGES‐RS Imaging Cardiac Evaluation to Locate Areas of Necrosis and Detect MI (ICELAND‐MI)

	Total Brain Parenchyma		Gray Matter		White Matter	
	Beta* (95% CI)	*P* Value	Beta* (95% CI)	*P* Value	Beta* (95% CI)	*P* Value
LVEF						
Model 1	−5.1 (−11.4, 1.1)	0.11	−4.2 (−8.1, −0.3)	0.03	−0.6 (−3.6, 2.2)	0.65
Model 2	−1.8 (−5.6, 1.9)	0.34	−1.5 (−4.5, 1.4)	0.31	−0.5 (−2.4, 1.5)	0.62
LVSV						
Model 1	−7.5 (−11.5, −3.4)	<0.001	−6.1 (−8.6, −3.6)	<0.001	−1.4 (−3.2, 0.5)	0.15
Model 2	−4.4 (−6.9, −1.9)	<0.001	−3.7 (−5.7, −1.8)	<0.001	−1.1 (−2.4, 0.2)	0.09
CO						
Model 1	−8.7 (−4.4, −3.0)	0.002	−8.0 (−11.5, −4.4)	<0.001	−0.9 (−3.6, 1.8)	0.50
Model 2	−3.9 (−7.4, −0.4)	0.030	−3.9 (−6.7, −1.2)	0.005	−0.3 (−2.1, 1.5)	0.76

Model 1: adjusted for age and sex; Model 2: adjusted for age, sex, education, current smoker, intracranial volume, history of hypertension, diabetes mellitus, systolic blood pressure, prevalent coronary heart disease, stroke, total cholesterol, body mass index, atrial fibrillation. AGES‐RS indicates age, gene/environment susceptibility‐Reykjavik Study; CO, cardiac output; LVEF, left ventricular ejection fraction; LVSV, left ventricular stroke volume; MI, myocardial infarction.

* Betas represent change in the brain volumes for each 10% lower LVEF, 10 mL lower LVSV and 1 L/min lower CO.

**Table 4.** Brain Tissue Volumes in Tertiles of Cardiac Hemodynamic Measures: AGES‐RS Imaging Cardiac Evaluation to Locate Areas of Necrosis and Detect MI (ICELAND‐MI)

Cardiac Hemodynamics	Total Brain Volume Mean (SE)	*P* for Trend	Grey Matter Volume Mean (SE)	*P* for Trend	White Matter Volume Mean (SE)	*P* for Trend
Ejection fraction						
Low	1066.4 (7.9)	0.626	667.5 (6.2)	0.573	371.2 (4.0)	0.325
Middle	1068.4 (7.9)		670.0 (6.1)		369.7 (4.0)	
High	1064.5 (8.1)		666.8 (6.3)		367.9 (4.1)	
Stroke volume						
Low	1060.5 (7.8)	0.009	663.5 (6.1)	0.008	367.9 (4.0)	0.185
Middle	1069.7 (8.0)		670.4 (6.2)		370.4 (4.1)	
High	1074.1 (8.0)		674.4 (6.2)		372.2 (4.1)	
Cardiac output						
Low	1063.6 (7.9)	0.004	665.2 (6.1)	0.002	368.7 (4.0)	0.503
Middle	1065.4 (7.9)		668.2 (6.1)		370.0 (4.0)	
High	1076.8 (8.1)		676.7 (6.3)		371.4 (4.1)	

All analyses were adjusted for age, sex, education, current smoker, intracranial volume, history of hypertension, diabetes mellitus, systolic blood pressure, prevalent coronary heart disease, stroke, total cholesterol, body mass index, and atrial fibrillation. Ranges for tertiles of LVEF are low: 15.2% to 58.4%, middle: 58.5% to 65.2% and high: 65.3% to 85.3%. Ranges for tertiles of LVSV are low: 20.3 to 55.3 mL, middle: 55.4 to 66.5 mL and high: 66.5 to 166.9 mL. Ranges for tertiles of CO are low: 1.6 to 3.4 L/min, middle: 3.5 to 4.2 L/min and high: 4.3 to 11. AGES‐RS indicates age, gene/environment susceptibility‐Reykjavik Study; CO, cardiac output; LVEF, left ventricular ejection fraction; LVSV, left ventricular stroke volume; MI, myocardial infarction.

We found no associations between cardiac hemodynamic parameters and manifestations of cerebral small vessel disease (Table 5) (all *P*>0.05). This was the same for age‐ and sex‐adjusted models as well as multivariable adjusted models. In multivariable analyses lower LVEF was associated with worse performance in executive function (*P*<0.001) (Table 6). Lower LVSV was associated with worse performance on processing speed (*P*=0.043) and executive function (*P*<0.001) tests. Likewise, lower CO was associated with worse scores in processing speed (*P*=0.015) and executive function (*P*=0.003). Adjusted mean values of the cognitive scores in tertiles of LVEF, LVSV, and CO are presented in Table 7.

**Table 5.** Association of Cardiac Hemodynamics With Cerebral Small Vessel Disease: AGES‐RS Imaging Cardiac Evaluation to Locate Areas of Necrosis and Detect MI (ICELAND‐MI)

	WMH		Infarcts		Microbleeds	
	Beta* (95% CI)	*P* Value	OR* (95% CI)	*P* Value	OR* (95% CI)	*P* Value
LVEF						
Model 1	0.18 (−1.08, 1.46)	0.25	1.21 (1.01, 1.45)	0.039	0.94 (0.72, 1.22)	0.63
Model 2	0.86 (−0.40, 2.1)	0.18	1.16 (0.96, 1.41)	0.131	0.85 (0.64, 1.13)	0.27
LVSV						
Model 1	−0.01 (−0.83, 0.80)	0.97	1.06 (0.94, 1.20)	0.31	1.04 (0.88, 1.24)	0.61
Model 2	0.33 (−0.52, 1.17)	0.63	1.09 (0.96, 1.25)	0.18	1.04 (0.86, 1.25)	0.67
CO						
Model 1	−0.18 (−1.33, 0.97)	0.76	1.01 (0.85, 1.20)	0.92	1.21 (0.93, 1.58)	0.15
Model 2	−0.32 (−1.17, 0.52)	0.44	0.99 (0.82, 1.19)	0.91	1.17 (0.89, 1.55)	0.26

Model 1: adjusted for age and sex; Model 2: adjusted for age, sex, education, current smoker, history of hypertension, diabetes mellitus, systolic blood pressure, prevalent coronary heart disease, total cholesterol, body mass index, atrial fibrillation. AGES‐RS indicates age, gene/environment susceptibility‐Reykjavik Study; CO, cardiac output; LVEF, left ventricular ejection fraction; LVSV, left ventricular stroke volume; MI, myocardial infarction; WMH, White matter hyperintensities.

* Betas and odds ratios are presented for each 10% decrease in LVEF, 10 mL decrease in LVSV and 1 L/min decrease in CO.

**Table 6.** Association of Cardiac Hemodynamic Measures With Cognitive Functioning: AGES‐RS Imaging Cardiac Evaluation to Locate Areas of Necrosis and Detect MI (ICELAND‐MI)

	Memory		Processing Speed		Executive Function	
	Beta* (95% CI)	*P* Value	Beta* (95% CI)	*P* Value	Beta* (95% CI)	*P* Value
LVEF						
Model 1	0.02 (−0.04, 0.07)	0.52	−0.05 (−0.10, −0.01)	0.06	−0.11 (−0.16, −0.06)	<0.001
Model 2	0.04 (−0.01, 0.10)	0.12	−0.02 (−0.06, 0.03)	0.48	−0.10 (−0.15, −0.05)	<0.001
LVSV						
Model 1	−0.02 (−0.06, −0.01)	0.18	−0.04 (−0.07, −0.01)	0.019	−0.05 (−0.08, −0.02)	0.001
Model 2	−0.02 (0.01, −0.06)	0.32	−0.03 (−0.06, −0.01)	0.043	−0.06 (−0.09, −0.03)	<0.001
CO						
Model 1	−0.01 (−0.05, 0.04)	0.84	−0.03 (−0.08, −0.01)	0.013	−0.05 (−0.09, −0.01)	0.013
Model 2	0.00 (−0.05, 0.05)	0.99	−0.03 (−0.07, −0.01)	0.015	−0.06 (−0.11, −0.02)	0.003

Model 1: adjusted for age and sex; Model 2: adjusted for age, sex, education, current smoker, history of hypertension, diabetes mellitus, systolic blood pressure, prevalent coronary heart disease, stroke, total cholesterol, body mass index, atrial fibrillation. AGES‐RS indicates age, gene/environment susceptibility‐Reykjavik Study; CO, cardiac output; LVEF, left ventricular ejection fraction; LVSV, left ventricular stroke volume; MI, myocardial infarction.

* Betas represent change in the brain volumes for each 10% lower LVEF, 10 mL lower LVSV and 1 L/min lower CO.

**Table 7.** Cognitive Function Scores in Tertiles of Cardiac Hemodynamic Parameters: AGES‐RS Imaging Cardiac Evaluation to Locate Areas of Necrosis and Detect MI (ICELAND‐MI)

Cardiac Hemodynamics	Memory Function Mean (SE)	*P* for Trend	Processing Speed Mean (SE)	*P* for Trend	Executive Function Mean (SE)	*P* for Trend
Ejection fraction						
Low	0.016 (0.12)	0.254	−0.073 (0.10)	0.965	−0.150 (0.10)	0.009
Middle	−0.031 (0.12)		−0.085 (0.10)		−0.017 (0.10)	
High	−0.092 (0.12)		−0.086 (0.10)		−0.008 (0.10)	
Stroke volume						
Low	−0.043 (0.12)	0.805	−0.147 (0.10)	0.042	−0.169 (0.10)	<0.001
Middle	−0.003 (0.12)		−0.048 (0.10)		−0.015 (0.10)	
High	−0.031 (0.12)		−0.011 (0.10)		0.055 (0.10)	
Cardiac output						
Low	−0.035 (0.12)	0.621	−0.111 (0.10)	0.051	−0.154 (0.10)	0.002
Middle	−0.012 (0.12)		−0.013 (0.10)		0.016 (0.10)	
High	−0.046 (0.12)		−0.065 (0.10)		0.007 (0.10)	

All analyses were adjusted for age, sex, education, current smoker, history of hypertension, diabetes mellitus, systolic blood pressure, prevalent coronary heart disease, stroke, total cholesterol, body mass index, and atrial fibrillation. Ranges for tertiles of LVEF are low: 15.2% to 58.4%, middle: 58.5% to 65.2% and high: 65.3% to 85.3%. Ranges for tertiles of LVSV are low: 20.3 to 55.3 mL, middle: 55.4 to 66.5 mL and high: 66.5 to 166.9 mL. Ranges for tertiles of CO are low: 1.6 to 3.4 L/min, middle: 3.5 to 4.2 L/min and high: 4.3 to 11.7 L/min. AGES‐RS indicates age, gene/environment susceptibility‐Reykjavik Study; CO, cardiac output; LVEF, left ventricular ejection fraction; LVSV, left ventricular stroke volume; MI, myocardial infarction.

In age‐ and sex‐adjusted analyses, lower LVEF, LVSV, and CO were not associated with depressive symptoms (Table 8). Further adjustment for cardiovascular risk factors did not change the associations. We observed that each 10 mL lower LVSV was associated with 1.24‐fold (95% CI 0.99 to 1.57) higher risk of cognitive impairment and each unit lower CO was associated with 1.40‐fold (95% CI 0.99 to 2.00) higher risk of cognitive impairment. All these associations approached statistical significance independent of socio‐demographic and cardiovascular factors.

**Table 8.** Association of Cardiac Hemodynamic Measures With Depression and cognitive impairment: AGES‐RS Imaging Cardiac Evaluation to Locate Areas of Necrosis and Detect MI (ICELAND‐MI)

	Depression		Cognitive Impairment*	
	OR* (95% CI)	*P* Value	OR* (95% CI)	*P* Value
LVEF				
Model 1	1.11 (0.81, 1.51)	0.49	1.17 (0.88, 1.56)	0.25
Model 2	1.10 (0.77, 1.56)	0.60	1.02 (0.75, 1.38)	0.89
LVSV				
Model 1	0.93 (0.76, 1.16)	0.52	1.28 (1.03, 1.58)	0.026
Model 2	0.86 (0.68, 1.09)	0.22	1.24 (0.99, 1.57)	0.070
CO				
Model 1	0.96 (0.64, 1.14)	0.29	1.51 (1.07, 2.12)	0.018
Model 2	0.79 (0.57, 1.07)	0.13	1.40 (0.99, 2.00)	0.067

Model 1: adjusted for age and sex; Model 2: adjusted for age, sex, education, current smoker, history of hypertension, diabetes mellitus, systolic blood pressure, prevalent coronary heart disease, stroke, total cholesterol, body mass index, atrial fibrillation. AGES‐RS indicates age, gene/environment susceptibility‐Reykjavik Study; CO, cardiac output; LVEF, left ventricular ejection fraction; LVSV, left ventricular stroke volume; MI, myocardial infarction.

* Defined as mild cognitive impairment or dementia.

* Betas and odds ratios are presented for each 10% lower LVEF, 10 mL lower LVSV and 1 L/min lower CO.

To test whether the association of cardiac hemodynamic parameters with brain volumes and cognitive functioning was the same in the groups of randomly selected subjects and diabetic patients, we performed a series of stratified analyses and found similar associations (all *P* for interactions >0.05) (Tables 9 and 10). In a sensitivity analysis, we excluded participants who had significant carotid stenosis (n=9) and observed that the associations remained essentially unchanged (data not shown). In addition, we excluded subjects in the lowest decile of the cardiac hemodynamic parameters and observed that the associations between hemodynamic parameters and features of brain aging did not essentially change (data not shown).

**Table 9.** Association of Cardiac Hemodynamic Measures With Brain Volumes in 2 Groups of Diabetics and Randomly Selected Subjects: AGES‐RS Imaging Cardiac Evaluation to Locate Areas of Necrosis and Detect MI (ICELAND‐MI)

	Total Brain Parenchyma		Gray Matter		White Matter	
	Beta* (95% CI)	*P* Value	Beta* (95% CI)	*P* Value	Beta* (95% CI)	*P* Value
LVEF						
Diabetics	2.1 (−7.6, 11.9)	0.66	−1.9 (−8.3, 4.4)	0.54	2.0 (−2.4, 6.4)	0.36
Randomly selected	−8.2 (−15.6, −0.8)	0.03	−5.4 (−10.1, −0.8)	0.02	−2.7 (−6.2, 0.8)	0.13
LVSV						
Diabetes	−9.7 (−16.6, −2.8)	0.006	−8.1 (−12.5, −3.6)	<0.001	−1.3 (−4.4, 1.9)	0.43
Randomly selected	−8.4 (−13.2, −3.6)	0.001	−6.5 (−9.5, −3.6)	<0.001	−2.1 (−4.3, 0.2)	0.07
CO						
Diabetics	−13.5 (−16.1, −2.6)	0.007	−10.9 (−17.6, −4.1)	0.002	−1.1 (−5.9, 3.6)	0.64
Randomly selected	−9.3 (−15.9, −4.5)	0.011	−8.4 (−12.5, −4.2)	<0.001	−1.5 (−4.1, 3.6)	0.37

All analyses were adjusted for age and sex. AGES‐RS indicates age, gene/environment susceptibility‐Reykjavik Study; CO, cardiac output; LVEF, left ventricular ejection fraction; LVSV, left ventricular stroke volume; MI, myocardial infarction.

* Betas represent change in the brain volumes for each 10% decrease in LVEF, 10 mL decrease in LVSV and 1 L/min decrease in CO.

**Table 10.** Association of Cardiac Hemodynamic Measures With Cognitive Functioning in 2 Groups of Diabetics and Randomly Selected Subjects: AGES‐RS Imaging Cardiac Evaluation to Locate Areas of Necrosis and Detect MI (ICELAND‐MI)

	Memory		Processing Speed		Executive Function	
	Beta* (95% CI)	*P* Value	Beta* (95% CI)	*P* Value	Beta* (95% CI)	*P* Value
LVEF						
Diabetics	−0.06 (−0.14, 0.02)	0.15	−0.06 (−0.11, 0.01)	0.15	−0.04 (−0.11, 0.03)	0.26
Randomly selected	0.02 (−0.05, 0.09)	0.55	−0.05 (−0.15, 0.02)	0.12	−0.12 (−0.18, −0.06)	<0.001
LVSV						
Diabetes	−0.07 (−0.13, −0.02)	0.014	−0.10 (−0.16, −0.03)	0.002	−0.07 (−0.12, −0.02)	0.007
Randomly selected	−0.03 (−0.07, 0.02)	0.21	−0.04 (−0.07, 0.00)	0.07	−0.05 (−0.09, −0.02)	0.005
CO						
Diabetics	−0.10 (−0.19, −0.01)	0.03	−0.12 (−0.21, −0.04)	0.005	−0.12 (−0.19, −0.04)	0.002
Randomly selected	−0.01 (−0.08, 0.05)	0.65	−0.04 (−0.09, 0.01)	0.16	−0.06 (−0.11, −0.05)	0.03

All analyses were adjusted for age and sex. AGES‐RS indicates age, gene/environment susceptibility‐Reykjavik Study; CO, cardiac output; LVEF, left ventricular ejection fraction; LVSV, left ventricular stroke volume; MI, myocardial infarction.

* Betas represent change in the brain volumes for each 10% increase in LVEF, 10 mL increase in LVSV and 1 L/min increase in CO.

## Discussion

Findings of this study suggest that a graded decrease in cardiac functioning, as reflected in cardiac hemodynamics, is associated with lower brain volumes and cognitive performance. The associations between cardiac hemodynamics and brain measures were independent of socio‐demographic and cardiovascular factors.

An increasing body of evidence indicates that patients with advanced heart failure carry a higher risk for manifestations of brain aging including gray matter loss, white matter hyperintensities, infarcts, and cognitive impairment.^9,27–29^ Recently, it has been proposed that not only subjects with advanced heart failure but also those with suboptimal cardiac functioning might be at higher risk for accelerated brain aging.^8^ A limited number of studies tested this hypothesis in community‐dwelling older adults. In a cohort of individuals from the Framingham Offspring Study with a mean age of 61 years, Jefferson et al showed that higher cardiac index was associated with higher total brain volume but they could not demonstrate a clear association with most of the neuropsychological measures acquired in the study.^12^ However, the interpretation of those findings is hampered because brain outcomes were assessed about 4 years before cardiac MRI measurements. On average, that cohort is also younger than our study population. In an another study, middle aged to elderly subjects in the lowest and highest quintile of the LVEF showed worse performance in memory and executive functioning as well as visuoperceptual abilities compared with the reference groups.^13^ In our cohort, with a mean age of 76 years, we showed that lower cardiac hemodynamic parameters, in particular stroke volume and cardiac output, were strongly associated with structural brain changes including lower total brain volume and gray matter volume as well as functional brain abnormalities including worse performance in processing speed and executive functioning. Moreover, we showed that lower stroke volume and cardiac output are associated with higher risk of mild cognitive impairment or dementia. We observed that lower cardiac output, in contrast to lower stroke volume, was not associated with memory function. Given that cardiac output is calculated by stroke volume multiplied by heart rate, and heart rate decreases with increasing age, it is possible that in older subjects stroke volume has a better predictive value for brain outcomes and in particular for memory function. Consistent with our findings, Jefferson et al did not show an association between cardiac index, derived from cardiac output, and volume of hippocampus which is greatly involved in memory function.^12^

Different mechanisms may explain a link between impaired cardiac functioning and brain. One possibility is shared co‐morbidities and vascular risk factors.^30^ That could, on the one hand, impair cardiac functioning and on the other hand, lead to brain structural and functional abnormalities.^31^ Available evidence for the importance of early recognition and management of vascular risk factors such as hypertension in prevention of cardiovascular and cerebrovascular events support this notion.^32^ However, we observed that adjustment of the analyses for established vascular risk factors did not essentially change our findings except for depressive symptoms. It is also possible that the decline in cardiac functioning and brain aging are epiphenomenon and they are not causally related. While this possibility cannot be totally excluded, previous population‐based cohort studies on older people free of dementia showed that subjects with heart failure are at a significantly higher risk for developing dementia.^29^ As an alternative explanation, impaired cardiac functioning could result in a decreased cerebral blood flow and consequently long‐standing cerebral hypoperfusion, via neuronal loss and injury, put the brain at higher risk for structural and functional abnormalities.^33^ In line with this hypothesis, it has been shown that interventions to enhance left ventricular functioning improve cognitive performance in patients with heart failure.^10^ In this study we observed that lower cardiac functioning was not related with markers of brain small vessel pathologies. This finding might indicate that long‐term decreased cardiac functioning mainly influences neuronal energy homeostasis, which ultimately results in neuronal cell death and brain tissue loss. Although a role for cerebral hypoperfusion as a mediator in the association between cardiac functioning and brain aging seems plausible, further research is needed to confirm this hypothesis and we are not able to establish a causal relationship only based on our observational data.

This study has certain strengths and limitations. A relatively large cohort of community‐dwelling older individuals with available data on cardiac functioning assessed by cardiac MRI, which is a reliable method and less operator dependent modality compared with conventional echocardiography,^34^ can be marked as the main strengths of this study. In addition, we had an extended set of data on structural and functional features of brain aging. A possible limitation is that our study population consists of randomly selected individuals as well as a group of individuals with diabetes. However, adjustments of the analyses for diabetes mellitus minimally changed the associations and the stratified analyses showed that the association between cardiac hemodynamics and brain measures was similar in both groups. As another limitation, due to the cross‐sectional design of this study, it is not clear whether changes in cardiac hemodynamics preceded changes in brain measures. This highlights a need for future longitudinal studies to investigate whether disturbances in cardiac hemodynamics are also associated with progression of the structural and functional brain pathologies. Participants in this study were Caucasian, which may limit extrapolation of our findings to the other race/ethnic groups.

In conclusion, our findings suggest that suboptimal cardiac functioning, independent of conventional cardiovascular risk factors, is associated with structural and functional features of brain aging in community‐dwelling older subjects. Previously, we have shown that coronary artery calcification is associated with structural and functional features of brain aging.^35^ While coronary artery calcification is more reflective of vascular function and cumulative measure of atherosclerosis, in this study we focused on cardiac function. Findings of current study confirms that disturbances in cardiac hemodynamics independent of cardiovascular risk factors and co‐morbidities are linked with adverse brain outcomes and interventions directed to maintain cardiac function in old age might have implications for preservation of brain function and structure. The cross‐sectional associations suggest that elderly patients presenting with cardiac or cognitive signs and symptoms may have both cardiac and cerebral disease and should be evaluated accordingly. Further, since there is a wider formulary for treating cardiac problems than there is for cognitive problems, clinicians treating cardiac problems should monitor a patient's cognition along with cardiac function. This is consistent with recent recognition and recommendations by the AHA that cardiovascular risk factors lead to vascular cognitive impairment.^26^ Future longitudinal and experimental studies will unravel the temporality and pathogenic mechanisms behind this association.
